# *STAT4* Gene Polymorphisms Are Associated with Susceptibility and ANA Status in Primary Biliary Cirrhosis

**DOI:** 10.1155/2014/727393

**Published:** 2014-02-04

**Authors:** Satoru Joshita, Takeji Umemura, Minoru Nakamura, Yoshihiko Katsuyama, Soichiro Shibata, Takefumi Kimura, Susumu Morita, Michiharu Komatsu, Akihiro Matsumoto, Kaname Yoshizawa, Hiromi Ishibashi, Eiji Tanaka, Masao Ota

**Affiliations:** ^1^Division of Gastroenterology and Hepatology, Department of Medicine, Shinshu University School of Medicine, 3-1-1 Asahi, Matsumoto 390-8621, Japan; ^2^Clinical Research Center, NHO Nagasaki Medical Center and Department of Hepatology, Nagasaki University Graduate School of Biomedical Sciences, 2-1001-1 Kubara, Omura 856-8562, Japan; ^3^Department of Pharmacy, Shinshu University Hospital, 3-1-1 Asahi, Matsumoto 390-8621, Japan; ^4^Department of Legal Medicine, Shinshu University School of Medicine, 3-1-1 Asahi, Matsumoto 390-8621, Japan

## Abstract

Recent genome-wide association studies suggest that genetic factors contribute to primary biliary cirrhosis (PBC) susceptibility. Although several reports have demonstrated that the interleukin (IL) 12 signaling pathway is involved in PBC pathogenesis, its precise genetic factors have not been fully clarified. Here, we performed an association analysis between *IL12A*, *IL12RB*, and *signal transducer and activator of transcription 4 (STAT4)* genetic variations and susceptibility to PBC. Single nucleotide polymorphisms (SNPs) were genotyped in 395 PBC patients and 458 healthy subjects of Japanese ethnicity and evaluated for associations with PBC susceptibility, anti-nuclear antibody (ANA) status, and anti-mitochondrial antibody (AMA) status. We detected significant associations with PBC susceptibility for several *STAT4* SNPs (rs10168266; *P* = 9.4 × 10^−3^, rs11889341; *P* = 1.2 × 10^−3^, rs7574865; *P* = 4.0 × 10^−4^, rs8179673; *P* = 2.0 × 10^−4^, and rs10181656; *P* = 4.2 × 10^−5^). Three risk alleles (rs7574865; *P* = 0.040, rs8179673; *P* = 0.032, and rs10181656; *P* = 0.031) were associated with ANA status, but not with AMA positivity. Our findings confirm that *STAT4* is involved in PBC susceptibility and may play a role in ANA status in the Japanese population.

## 1. Introduction

Primary biliary cirrhosis (PBC) is an autoimmune liver disease characterized by destruction of intrahepatic bile ducts and development of hepatic fibrosis that often progress to cirrhosis and liver failure [1]. The etiology of PBC remains poorly understood and is considered to be complex, [2–4] whereby a combination of inherited genetic predisposition factors and environmental exposure is likely required for disease development. Several genetic characteristics have specifically been implicated in PBC etiology in the Japanese population, including the HLA DRB1*08 : 03-DQB1*06 : 01 haplotype and single nucleotide polymorphisms (SNPs) in the *cytotoxic T-lymphocyte-associated protein 4 *and* ataxin 2-binding protein 1* genes [5–7].

Recent genome-wide association studies (GWAS) have identified a number of HLA and non-HLA loci with possible relevance to the development of PBC. However, these studies often uncovered different loci in the same signaling pathways across ethnicities; [8–12] genetic variants of the *IL12 *α*-chain* (*IL12A*) and *IL12RB2* genes were associated with disease susceptibility in Caucasian studies, [8–11] but such associations have not been confirmed in the Japanese [12].

Signal transducer and activator of transcription 4 (STAT4) is a transcription factor belonging to the STAT family [13] that is required for the development of Th1 cells from naïve CD4+ T cells [14] and IFN-*γ* production in response to IL12 [15]. Two chains of the IL12 receptor form a heterodimer after IL12 binding and activate the receptor-associated JAK kinases JAK2 and TYK2. STAT4 is phosphorylated by these tyrosine kinases, homodimerizes via its src homology 2 (SH2) domain, and then translocates into the nucleus to activate cytokine-responsive gene transcription [16]. While early GWAS initially showed a weak association between *STAT4* polymorphisms and PBC susceptibility, [8–10] recent investigations have confirmed a definite link between the two [11, 12] and have indicated that common pathogenic pathways, such as IL12 signaling, play an essential and nonredundant role in the development of this disease and some of its clinical features.

Anti-mitochondrial antibody (AMA) positivity is the serologic hallmark of PBC. AMA titers tend to be stable over time in individual patients and do not correlate with disease severity or rate of progression [1, 17]. Antinuclear antibodies (ANA) are found in up to 70% of patients with PBC and are suggested to be associated with more rapid disease progression and a poorer prognosis [18]. Positivity for anti-gp210 and anti-centromere antibodies has been related to PBC progression as well [19, 20]. Since the association between genetic polymorphisms and autoantibody production has not yet been elucidated, we investigated whether such polymorphisms contributed to a genetic predisposition to PBC and autoantibody production in the Japanese population.

## 2. Patients and Methods

### 2.1. Ethics Statement

This study was approved by the ethics committees of both participating institutions (Shinshu University School of Medicine, Matsumoto, Japan, and the National Hospital Organization Nagasaki Medical Center, Omura, Japan), and written informed consent was obtained from all participants. The study was conducted in accordance with the principles of the Declaration of Helsinki.

### 2.2. Subjects

We analyzed a total of 853 subjects (395 PBC patients and 458 sex-matched healthy controls) enrolled at Shinshu University Hospital, Matsumoto, Japan, and the National Hospital Organization Nagasaki Medical Center, Omura, Japan. As the subjects had no direct relatives of non-Japanese ethnicity, their racial background was considered to be uniformly Japanese. A part of this study's participants had been enrolled in previous genetic association studies [5–7, 12, 21–25]. In particular, 298 of 395 patients (75.4%) had been included in an earlier GWAS from Japan and were defined as the GWAS cohort in this analysis [12]. The remaining 97 (24.6%) patients were newly diagnosed as having PBC and were defined as the replication cohort. Newly enrolled control subjects were volunteers from hospital staff who had indicated the absence of any major illnesses in a standard questionnaire and whose racial background was considered to be uniformly Japanese. The sex-matched control group consisted of 384 women and 74 men with no direct familial relations.

The diagnosis of PBC was determined based on criteria from the American Association for the Study of Liver Diseases [26]. Serum AMA, which is specific for the pyruvate dehydrogenase complex-E2 component, was measured by the enzyme-linked immunosorbent assay (ELISA). An index of greater than 7 was considered to be a positive result. Serum ANA was determined by immunofluorescence using HEp-2 cells, whereby a titer of ≥40 was considered to be positive, as reported previously [27]. Patterns of ANA reactivity were recorded as well. Serum anti-centromere antibody was detected using commercially available ELISA kits (MBL, Nagoya, Japan), for which an index of >5 U/mL was considered to be positive. Serum gp-210 antibody was also measured by ELISA, whereby an index of >10 U/mL was considered to be a positive result, as previously described [19]. All patients were negative for hepatitis B surface antigen and antibodies to the hepatitis C and human immunodeficiency viruses.

### 2.3. IL-12 Signaling-Related SNP Genotyping

Genomic DNA from patients was isolated by phenolic extraction of sodium dodecyl sulfate-lyzed and proteinase K-treated cells, as described previously [28], and adjusted to a concentration of 10–15 ng/*μ*L.

*IL12A *(rs574808)*, IL12RB *(rs3790567), and* STAT4* (rs7574865) SNPs were selected based on reported PBC susceptibility [8–12]. Since the *STAT4* (rs7574865) SNP was found to be significantly associated with PBC, we genotyped an additional 7 SNPs located in this gene (rs10168266, rs7594501, rs16833239, rs11889341, rs8179673, rs10181656, and rs6752770) that were not evaluated in the earlier Japanese GWAS using an SNP Genotyping Kit (Applied Biosystems, Tokyo, Japan). These SNPs were selected based on previous reports [8–12, 29–31]. Polymerase chain reaction (PCR) was performed with TaqMan Assays (7500 Real Time PCR System; Applied Biosystems, Foster City, California, USA) following the manufacturer's instructions.

### 2.4. Statistical Analysis

All examined SNPs in control groups were in the Hardy-Weinberg equilibrium. The R-software “Haploview” [32] version 4.2 was used to evaluate the haplotype structure of the 8 *STAT4* SNPs. Pairwise linkage disequilibrium (LD) patterns and haplotype frequency analysis for all SNPs in patients and controls were assessed by the block definition established by Gabriel et al. [33]. We assessed the significance of allele distribution between patients and controls using the *χ* ^2^ test by means of 2 × 2 comparisons. A *P* value of less than 0.05 was considered to be statistically significant. We adjusted *P* values using Bonferroni's correction by multiplying each locus by 8 (*P*_*c*_). Association strength was estimated by calculating the odds ratio (OR) and 95% confidence interval (CI). Statistical analysis of data was performed using SPSS 21.0 software (IBM, Armonk, New York).

## 3. Results

### 3.1. Genotyping of IL12 Signaling-Related SNPs

To clarify the genetic susceptibility to PBC based on previously reported *IL12* signaling, a total of 395 Japanese patients with PBC and 458 healthy Japanese controls were enrolled for an association analysis of *IL12A *(rs574808)*, IL12RB *(rs3790567), and* STAT4* (rs7574865) SNPs (Table 2). Whereas the *IL12A* and *IL12RB* SNPs were not associated with PBC, the rs7574865 SNP in *STAT4* showed a positive association with PBC susceptibility (41.9% versus 33.5%; *P* = 4.0 × 10^−4^, OR = 1.43, 95% CI = 1.17–1.74). To further examine its role in PBC, we selected an additional 7 SNPs from *STAT4* (rs10168266, rs7594501, rs16833239, rs11889341, rs8179673, rs10181656, and rs6752770) and genotyped them in all patients and controls. The minor allele frequencies of A at rs10168266, T at rs11889341, T at rs7574865, G at rs8179673, and G at rs10181656 were significantly increased in PBC patients as compared with controls (*P* = 9.4 × 10^−3^, *P* = 1.2 × 10^−3^, *P* = 4.0 × 10^−4^, *P* = 2.0 × 10^−4^, and *P* = 4.0 × 10^−5^, resp.) (Table 3).

### 3.2. Distribution of *STAT4* Haplotypes among PBC Patients and Controls

**Figure 1:**
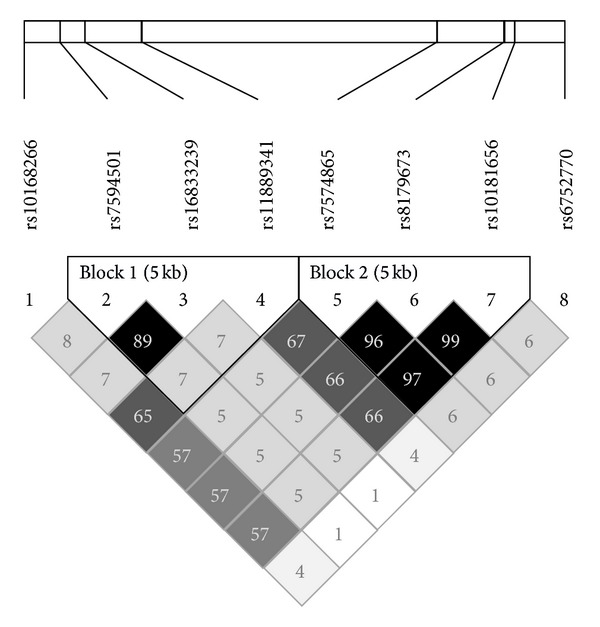
Linkage disequilibrium (LD) plot of 8 SNPs in *STAT4* in 458 healthy subjects. Values of *r* ^2^ corresponding to each SNP pair are expressed as a percentage and shown within the respective square.

We firstly defined LD blocks for the 8 SNPs in *STAT4* (Figure 1). The *STAT4* region was divided into two haplotype blocks, with substantial LD among the SNPs in each block. To estimate haplotype frequency and analyze haplotype association with PBC, we selected tag SNPs using the Tagger algorithm from the Haploview program. Three tag SNPs (rs7594501, rs16833239, and rs11889341) in block A and 3 tag SNPs (rs7574865, rs8179673, and rs10181656) in block B were captured from pairwise measures of LD. The top 3 haplotype frequencies in both blocks are shown in Table 4. Haplotype 2 (GCT) in block A was significantly associated with PBC susceptibility (40.3% versus 32.2%; *P* = 3.5 × 10^−3^, OR 1.43, 95% CI 1.12–1.81), as was haplotype 5 (TGG) in block B (43.3% versus 33.6%; *P* = 6.0 × 10^−4^, OR 1.51, 95% CI 1.19–1.91). In contrast, protective effects were seen for haplotype 4 (GAC) in block B (53.9% versus 65.4%; *P* = 5.0 × 10^−5^, OR 0.62, 95% CI 0.49–0.78).

### 3.3. Associations between *STAT4* SNPs, Haplotypes, and Autoantibodies

The PBC patients enrolled in this study were highly positive for disease-specific autoantibodies (Table 1). There were no significant differences with regard to ANA positivity between 250 of 369 AMA-positive patients (67.8%) and 21 of 26 AMA-negative patients (80.8%) (*P* = 0.245). Among the 8 *STAT4 *SNPs, the frequencies of 3 minor alleles (T at rs7574865, G at rs8179673, and G at rs10181656) were increased in ANA-positive PBC patients as compared with ANA-negative patients (*P* = 0.040, *P* = 0.032, and *P* = 0.031, resp.) (Table 5), but these statistical significances disappeared after correction. Haplotype 5 (TGG) in block B was significantly correlated with ANA (44.3% versus 36.3%; *P* = 0.035, OR 1.40, 95% CI 1.02–1.90) (Table 6). Interestingly, haplotype 5 was also significantly associated with the speckled pattern of ANA as compared with the nonspeckled pattern (45.8% versus 36.3%; *P* = 0.029, OR 1.48, 95% CI 1.04–2.12) (data not shown). No *STAT4* SNPs or haplotypes were associated with other autoantibody positivity or ANA pattern, such as discrete speckled pattern, homogenous pattern, nucleolar pattern, or peripheral pattern.

## 4. Discussion

In the present study, we investigated the association between *STAT4 *SNPs and PBC susceptibility and its clinical significance in the Japanese population. Our key findings were as follows: (1) specific *STAT4* polymorphisms and haplotypes were significantly associated with PBC susceptibility or protection; (2) there were no significant genetic associations between *IL12A* and* IL12RB* SNPs and PBC susceptibility, in contrast to studies of Caucasians; [8–11] and (3) there was a moderate relationship between *STAT4* SNPs and ANA-positive, but not AMA-positive, PBC patients.

STAT4 lies in the signaling pathways of several cytokines, such as IL12, type I interferon, and IL23. This time, a newly diagnosed PBC cohort, albeit small, replicated the previous finding by Japanese GWAS [12] that a *STAT4* SNP at rs7574865 was associated with susceptibility to PBC. To evaluate the power of this study, larger association studies of other ethnicities, including Chinese and Caucasian populations, are required because small sample sizes may lead to false positive or negative results. Moreover, we identified 4 additional SNPs in *STAT4* that conferred susceptibility to PBC and were consistent with findings of GWAS of other ethnicities [11]. Haplotype analysis showed that 3 of the identified risk SNPs, rs7574865, rs8179673, and rs10181656, were located in the same LD block (Figure 1 and Table 4). These SNPs and this haplotype have been linked with several autoimmune diseases, including rheumatoid arthritis and systemic lupus erythematosus (SLE) [29–31, 34–38]. In particular, it has been reported that SNPs at rs7574865 are associated with numerous other autoimmune diseases [35–38]. As we focused only on *STAT4* polymorphisms, we must concede that our study is rather limited in depth and scope compared with recent immunogenic studies [39, 40] using the Immunochip. However, our findings support the notion that SNPs and haplotypes in *STAT4* may contribute to the development of PBC and other autoimmune diseases.

In addition to our own, two earlier studies from Japan [12, 41] showed that *IL12A *and *IL12RB *SNPs were not associated with PBC, which was in contrast to strong associations found in Caucasian population studies [8–10, 42]. Similarly, GWAS of the Japanese have identified novel significant susceptibility loci for PBC, such as *TNFSF15 *and *POU2AF1*, which have not been identified in GWAS of populations of European descent. Meanwhile, Peters et al. found that liver damage severity at clinical presentation is higher among non-Caucasians than Caucasians for PBC [43]. Hence, although the IL12 pathway through STAT4 plays an essential role in PBC etiology, there is evidence of ethnic differences in genetic susceptibility loci. PBC is also concurrent with other autoimmune diseases, including Sjögren syndrome, [44] rheumatoid arthritis, [45] and cutaneous scleroderma, [46] so we cannot exclude the possibility of genetic overlap among these disorders.

Interestingly, our study showed a moderate association between 3 *STAT4* polymorphisms and 2 haplotypes with ANA-positive PBC that was not seen for AMA (Tables 5 and 6). To understand its clinical relevance, we analyzed this association with regard to ANA pattern and found a significant relationship between these SNPs and the speckled pattern of ANA, which was in agreement with a recent meta-analysis showing that the presence of anti-ds-DNA antibodies was associated with rs7574865 within *STAT4* polymorphisms in SLE patients. Our results did not support a relationship between *STAT4* SNPs and gp210 or anti-centromere antibodies, despite these antibodies having been associated with disease progression and prognosis in PBC patients [19]. Taken together, our data implied that *STAT4* SNPs imparted susceptibility to ANA-positive PBC, but for reasons that are still unknown. The mechanisms by which genetic variants are correlated with ANA positivity may be diverse and require further study.

## 5. Conclusions

Our findings confirm that *STAT4* SNPs and haplotypes contribute to PBC susceptibility and may play a role in mediating ANA status. *STAT4* appears to factor strongly in the pathogenesis of this and other autoimmune diseases and requires continued study.

## Figures and Tables

**Table 1:** Demographic and clinical data of patients.

Characteristic	*n* = 395
Median age, years (range)	58 (28–87)
Female/male	338/57
Autoantibody	
AMA-positive, *n* (%)	369 (93.4)
ANA-positive, *n* (%)	271 (67.6)
Cenp positive, *n* (%)	119/362 (32.9)*
gp210 positive, *n* (%)	80/260 (30.8)*

AMA: anti-mitochondrial antibody; ANA: anti-nuclear antibody; Cenp: anti-centromere antibody; gp210: gp210 antibody.

*Only patients who were assessed for Cenp and gp210 are reported.

**Table 2:** *IL12A*, *IL12RB*, and *STAT4* SNPs in PBC patients and healthy subjects.

Gene	db SNP	Allele	GWAS cohort	Replication cohort	Combined cohort				
		minor/major	patients (%)	patients (%)	patients (%)	Controls (%)	*P *	OR	95% CI
			(*n* = 302)	(*n* = 93)	(*n* = 395)	(*n* = 458)			
*IL12A *	rs574808	C/T	17.2	18.3	17.5	18.1	0.75		
*IL12RB *	rs3790567	A/G	26.8	22.0	25.7	22.9	0.18		
*STAT4 *	rs7574865	T/G	40.6	46.8	41.9	33.5	4.0 × 10^−4^	1.43	1.17–1.74

*IL12*: interleukin 12; *STAT4*: signal transducer activator transcription 4; PBC: primary biliary cirrhosis; SNP: single nucleotide polymorphism; OR: odds ratio; CI: confidence interval.

**Table 3:** *STAT4* SNPs in PBC patients and healthy subjects.

db SNP	Allele minor/major	Patients (%) (*n* = 395)	Controls (%) (*n* = 458)	*P *	*P*_*c*_	OR	95% CI
rs10168266	A/G	34.5	28.7	9.4 × 10^−3^	0.038	1.31	1.07–1.61
rs7594501	A/G	12.7	16.7	0.021			
rs16833239	T/C	13.0	16.4	0.046			
rs11889341	T/C	39.1	31.7	1.2 × 10^−3^	9.6 × 10^−3^	1.39	1.14–1.69
rs7574865	T/G	41.9	33.5	4.0 × 10^−4^	3.2 × 10^−3^	1.43	1.17–1.74
rs8179673	G/A	42.1	33.5	2.0 × 10^−4^	1.6 × 10^−3^	1.44	1.19–1.75
rs10181656	G/C	43.3	33.7	4.2 × 10^−5^	3.4 × 10^−4^	1.50	1.24–1.83
rs6752770	G/A	15.0	17.8	0.120			

*STAT4*: signal transducer activator transcription 4; PBC: primary biliary cirrhosis; SNP: single nucleotide polymorphism; OR: odds ratio; *P*_*c*_: corrected* P *value; CI: confidence interval.

**Table 4:** *STAT4* haplotypes in PBC patients and healthy subjects.

Block	Haplotype		SNPs		Patients (%)	Controls (%)	*P *	OR	95% CI
					(n* = 790)	(n* = 916)			
A		rs7594501	rs16833239	rs11889341					
	1	G	C	C	46.4	50.0	0.222		
	2	G	C	T	40.3	32.2	3.5 × 10^−3^	1.43	1.12–1.81
	3	A	T	C	11.9	15.9	0.045		

B		rs7574865	rs8179673	rs10181656					
	4	G	A	C	53.9	65.4	5.0 × 10^−5^	0.62	0.49–0.78
	5	T	G	G	43.3	33.6	6.0 × 10^−4^	1.51	1.19–1.91
	6	G	A	G	2.1	0.0	3.0 × 10^−4^		

*STAT4*: signal transducer activator transcription 4; PBC: primary biliary cirrhosis; SNP: single nucleotide polymorphism; OR: odds ratio; CI: confidence interval; n*: values for n* indicate two times the number of individuals since each person carries two haplotypes.

**Table 5:** Correlations between *STAT4 *SNPs and autoantibody positivity.

db SNP	Allele	ANA^+^ (%)	ANA^−^ (%)	*P *	OR	95% CI	AMA^+^ (%)	AMA^−^ (%)	*P *	Cenp^+^ (%)	Cenp^−^ (%)	*P *	gp210^+^ (%)	gp210^−^ (%)	*P *
	minor/major	(n* = 271)	(n* = 124)				(n* = 369)	(n* = 26)		(n* = 119)	(n* = 243)		(n* = 80)	(n* = 180)	
rs10168266	A/G	35.6	33.1	0.50			34.2	42.3	0.24	36.1	35.3	0.83	35.0	32.5	0.58
rs7594501	A/G	11.5	14.9	0.18			12.9	7.7	0.27	13.0	12.6	0.87	11.9	12.8	0.77
rs16833239	T/C	11.6	15.3	0.15			13.1	7.7	0.26	12.6	13.4	0.77	13.1	13.1	0.98
rs11889341	T/C	41.3	34.7	0.076			38.8	46.2	0.29	59.7	59.7	1.00	39.4	37.2	0.64
rs7574865	T/G	44.5	36.7	0.040	1.38	1.01–1.88	41.6	48.1	0.36	42.4	43.6	0.76	43.1	38.3	0.30
rs8179673	G/A	44.8	36.7	0.032	1.40	1.03–1.91	41.9	48.1	0.38	42.9	43.8	0.81	43.1	38.6	0.33
rs10181656	G/C	46.1	37.9	0.031	1.40	1.03–1.91	43.1	50.0	0.33	45.0	44.4	0.90	43.1	38.6	0.33
rs6752770	G/A	15.4	14.1	0.63			15.3	11.5	0.47	13.4	14.7	0.64	15.0	15.8	0.81

*STAT4*: signal transducer activator transcription 4; PBC: primary biliary cirrhosis; SNP: single nucleotide polymorphism; OR: odds ratio; CI: confidence interval; AMA: anti-mitochondrial antibody; ANA: anti-nuclear antibody; Cenp: anti-centromere antibody; gp210: gp210 antibody; n*: values for n* indicate two times the number of individuals since each person carries two haplotypes.

**Table 6:** Correlations between *STAT4 *haplotypes and autoantibody positivity.

Block	Haplotype		SNPs		ANA^+^ (%)	ANA^−^ (%)	*P *	OR	95% CI	AM^+^ (%)	AMA^−^ (%)	*P *	Cenp^+^ (%)	Cenp^−^ (%)	*P *	gp210^+^ (%)	gp210^−^ (%)	*P *
					(n* = 542)	(n* = 248)				(n* = 738)	(n* = 52)		(n* = 238)	(n* = 486)		(n* = 160)		
A		rs7594501	rs16833239	rs11889341														
	1	G	C	C	46.7	49.6	0.45			47.7	46.2	0.83	46.6	46.1	0.89	47.5	49.2	0.73
	2	G	C	T	41.3	34.7	0.076			38.8	46.2	0.29	40.3	40.3	1.00	39.4	37.2	0.64
	3	A	T	C	11.1	14.5	0.17			12.5	7.7	0.31	12.6	12.3	0.92	11.9	12.2	0.91

B		rs7574865	rs8179673	rs10181656							0.0							
	4	G	A	C	53.7	61.7	0.035	0.72	0.53–0.98	56.8	48.1	0.22	54.6	55.3	0.85	56.9	61.4	0.33
	5	T	G	G	44.3	36.3	0.035	1.40	1.02–1.90	41.5	46.2	0.51	42.0	43.4	0.72	43.1	38.3	0.30
	6	G	A	G	1.5	1.6	0.88			1.4	3.8	0.16	2.5	0.8	0.066	0.0	0.0	—

*STAT4*: signal transducer activator transcription 4; PBC: primary biliary cirrhosis; SNP: single nucleotide polymorphism; OR: odds ratio; CI: confidence interval; AMA: anti-mitochondrial antibody; ANA: anti-nuclear antibody; Cenp: anti-centromere antibody; gp210: gp210 antibody; n*: values for n* indicate two times the number of individuals since each person carries two haplotypes.

## Abbreviations

PBC: Primary biliary cirrhosis
SNP: Single nucleotide polymorphism
GWAS: Genome-wide association study
IL12: Interleukin12
STAT4: Signal transducer and activator of transcription 4
AMA: Anti-mitochondrial antibody
ANA: Anti-nuclear antibody
PBMCs: Peripheral blood mononuclear cells
LD: Linkage disequilibrium
*P*_*c*_: Corrected *P*
OR: Odds ratio
CI: Confidence interval.
